# The Genetic Basis of Strokes in Pediatric Populations and Insight into New Therapeutic Options

**DOI:** 10.3390/ijms23031601

**Published:** 2022-01-29

**Authors:** Milena Jankovic, Bojana Petrovic, Ivana Novakovic, Slavko Brankovic, Natasa Radosavljevic, Dejan Nikolic

**Affiliations:** 1 Neurology Clinic, Clinical Center of Serbia, 11000 Belgrade, Serbia; milena.jankovic.82@gmail.com; 2 Clinic of Gynecology and Obstetrics, Clinical Center of Serbia, 11000 Belgrade, Serbia; mrdrbojaninmail@gmail.com; 3 Faculty of Medicine, University of Belgrade, 11000 Belgrade, Serbia; ivana.novakovic@med.bg.ac.rs; 4 Faculty of Sciences and Mathematics, University of Priština in Kosovska Mitrovica, 38220 Kosovska Mitrovica, Serbia; slavko.brankovic@pr.ac.rs; 5 Department of Physical Medicine and Rehabilitation, King Abdulaziz Specialist Hospital, Taif 26521, Saudi Arabia; dr.natasa.radosavljevic@gmail.com; 6 Physical Medicine and Rehabilitation Department, University Children’s Hospital, 11000 Belgrade, Serbia

**Keywords:** stroke, children, genetics, diagnostics, treatment

## Abstract

Strokes within pediatric populations are considered to be the 10th leading cause of death in the United States of America, with over half of such events occurring in children younger than one year of life. The multifactorial etiopathology that has an influence on stroke development and occurrence signify the importance of the timely recognition of both modifiable and non-modifiable factors for adequate diagnostic and treatment approaches. The early recognition of a stroke and stroke risk in children has the potential to advance the application of neuroprotective, thrombolytic, and antithrombotic interventions and rehabilitation strategies to the earliest possible timepoints after the onset of a stroke, improving the outcomes and quality of life for affected children and their families. The recent development of molecular genetic methods has greatly facilitated the analysis and diagnosis of single-gene disorders. In this review, the most significant single gene disorders associated with pediatric stroke are presented, along with specific therapeutic options whenever they exist. Besides monogenic disorders that may present with stroke as a first symptom, genetic polymorphisms may contribute to the risk of pediatric and perinatal stroke. The most frequently studied genetic risk factors are several common polymorphisms in genes associated with thrombophilia; these genes code for proteins that are part of the coagulation cascade, fibrolysis, homocystein metabolism, lipid metabolism, or platelets. Single polymorphism frequencies may not be sufficient to completely explain the stroke causality and an analysis of several genotype combinations is a more promising approach. The recent steps forward in our understanding of the disorders underlying strokes has given us a next generation of therapeutics and therapeutic targets by which to improve stroke survival, protect or rebuild neuronal connections in the brain, and enhance neural function. Advances in DNA sequencing and the development of new tools to correct human gene mutations have brought genetic analysis and gene therapy into the focus of investigations for new therapeutic options for stroke patients.

## 1. Introduction

Strokes within the pediatric population are considered to be the 10th leading cause of death in the United States of America (USA), with over half of such events occurring in children younger than one year of life [1]. It is important to note that higher mortality rates (up to 25%) are for hemorrhagic stroke, while lower mortality rates (up to 10%) are associated with arterial ischemic stroke (AIS) [2]. 

The incidence of strokes in children ranges from around 2–3 per 100,000 births yearly, with the incidence being higher in neonates, ranging between 25–40 per 100,000 children, and even higher in premature neonates, with estimates around 100 per 100,000 children [3]. 

Strokes within the pediatric population can be classified as ischemic or hemorrhagic based on the cause of the stroke. Ischemic stroke can be further classified as AIS or venous infarction, where a venous infarction can be caused by cerebral sinovenous thrombosis (CSVT) or cortical vein thrombosis [4]. Furthermore, a stroke can be classified within the pediatric population according to the age of the patient: a perinatal stroke is a stroke that occurs between 28 weeks of gestation and 28 days of postnatal life, and a childhood stroke is classified as one occurring between 28 days of life and 18 years [4].

The aim of this review is to summarize and discuss recent knowledge regarding the classification, genetic basis, and therapeutic options of strokes within a pediatric population.

## 2. Arterial Ischemic Stroke in Children

In a pediatric population, the incidence of AIS is higher for those above 5, and up to 14, years of life (8–13 per 100,000 births) and lower for those below 5 years of life (2–3 per 100,000 births) in Western countries [5]. AIS constitutes around 80% of all strokes in the perinatal period, while in childhood, it ranges between 1–2 per 100,000 children in Western countries. In a previous review by Felling et al., it was noted that rates of perinatal AIS varied from 17.8 per 100,000 newborns in a National Hospital Discharge Survey to 13 symptomatic perinatal AIS per 100,000 newborns in a Swiss study conducted between 2000–2010 [6]. Furthermore, regarding childhood stroke, male children are more frequently affected, and a higher frequency of AIS occurs in Black and Asian children [4]. In the study carried out by Amlie-Lefond, it was stated that mortality following AIS ranged between 6–10% in children, while more than 75% of those who suffered AIS are being associated with long-term neurological deficits [7]. McKinney and colleagues have stressed that for more than half of children with AIS, there is an association with a decreased quality of life [3].

In pediatric populations, the risk factors associated with AIS are cardiac disorders, including congenital heart disease, infections, head and neck trauma, prothrombotic disorders, sickle cell disease, cancer, genetic disorders, and inborn errors of metabolism, as well as autoimmune and inflammatory states [8]. However, the most important risk factors for AIS in pediatric populations are arteriopathies, congenital and acquired cardiac diseases, thrombophilia, and prothrombotic disorders [9]. Furthermore, in the literature, risk factors for recurrent AIS were also described. They include arteriopathies, heart disease, prothrombotic risk factors (antithrombin deficiency, elevated lipoprotein (a), and more than one prothrombotic factor), clinical presentation without seizures, and genetic polymorphisms [9,10,11].

Data from the Canadian Pediatric Ischemic Stroke Registry that were published in the study of deVeber et al. pointed out that predictors of poor clinical outcome in neonates include an altered level of consciousness, non-specific presentation, and bazal ganglia involvement; in older children, these predictors include non-specific presentation, seizures, cardiac risk factor, arteriopathy risk factor, and no treatment [10]. 

In the study done by Andrade et al., it was stated that between 50–80% of children with AIS have at least one risk factor [5]. Moreover, in the perinatal period, it is likely that AIS etiology is multifactorial, with an increased risk when multiple risk factors are present [12].

### Pathophysiological Mechanisms of a Hypoxic-Ischemic Injury

After an arterial ischemic event within the perinatal period, a series of cellular and molecular actions begin, resulting in necrotic and apoptotic cell death [12]. Bearing in mind the complex interactions that result from an ischemic event and the crosstalk between systemic circulation and the brain via the blood–brain barrier, it should be stressed that, compared with adults, such a barrier is not permeable in the early newborn period after stroke [12]. The hypoxic-ischemic (HI) injury results in neuronal death in two phases [13]. The HI event leads to the anaerobic metabolism of cells due to the lack of energy substrates, oxygen, and glucose. This results in primary energy failure, an increased intracellular lactate production which elevates reactive oxygen species (ROS) levels, and, consequently, necrotic cell death. During the primary energy failure, there is an uncontrolled release of excitatory neurotransmitters, a disruption of the blood–brain barrier (BBB), and an inflammatory response activation; in particular, within minutes after the HI injury in immature brains, an innate immune response is activated [14]. If the insult persists, this will result in a secondary energy failure (hours (at least 6 h post injury) to days), where most cell deaths are due to apoptosis [12]. In this phase of excitotoxicity, oxidative stress, inflammation, and mitochondrial dysfunction are all observed [12,13]. It was demonstrated that inflammation by itself can induce neuronal cell death [15]. Furthermore, inflammation can trigger the release of excitotoxic molecules and influence the release of cytokines, free radicals, and other toxic elements that could lead to neuronal cell death as a result of neonatal brain injury [15]. Finally, after the secondary phase, there is a chronic phase that includes astrogliosis, chronic inflammation, and tissue repair and remodeling [13].

## 3. Cerebral Sinovenous Thrombosis in Children

In patients with CSVT, there is a cerebral blood flow disruption due to the occlusion of cerebral veins and/or sinuses [16]. Despite the fact that CSVT is not frequent within the neonatal period, it is associated with poor outcomes and significant mortality and morbidity, while older children mostly make a full recovery [16,17]. In Europe and North America, the incidence of CSVT in childhood is estimated to be around 0.6 per 100,000 individuals, where 30–50% of these cases are in neonates [17]. Cardiello and Rossi stated that the annual incidence of CSVT in neonates is 7 per 1 million newborns [16]. In children, like within the adult population, the superficial sinuses are most frequently involved, with the transverse sinuses being more frequently involved in children older than two years of life [18]. In the study conducted by Sellers et al., the independent risk factors for CSVT in children are head and neck trauma and infection, as well as mechanical ventilation [19]. Furthermore, hematological conditions, malignancy, congenital heart disease, and inflammatory bowel disease are described as predisposing factors to CSVT [20]. Moreover, Sorg et al. determined that hypoxia within the neonatal period was independently associated with CSVT [21]. 

## 4. Hemorrhagic Stroke in Children

In infants and children, most cases of non-traumatic subarachnoid hemorrhage (SAH) and intracerebral hemorrhage (ICH) are due to a secondary pathology [22]. The most common etiology of spontaneous ICH includes vascular malformations, bleeding diathesis, aneurysms, hemorrhagic primary intracranial tumors, and other pathologies including infections of central nervous system (CNS) [22]. For the group with intracranial aneurisms, certain heritable disorders could be associated with the increased incidence. Among these heritable disorders are those with autosomal dominant, autosomal recessive, and X-linked recessive inheritance [22]. In a study done by Huang and colleagues, for children aged between 7–14 years with spontaneous ICH, the most common cause of such an event were cerebrovascular disorders, while ICH presenting in infants and young children (29 days of life up to 3 years) were mostly associated with hematologic diseases [23].

### Pathophysiological Mechanisms of Brain Damage after Intracerebral Hemorrhage

In individuals with ICH, there are several mechanisms that cause brain damage. Among them, one is mechanical brain injury. There may be reduction in local cerebral blood flow and increased intracranial pressure that can lead to edema, which will contribute to the additional secondary brain damage [24]. 

Another mechanism refers to the complex immune, coagulation, and inflammatory cascades [24,25]. Xi et al. stated that the activation of te coagulation cascade plays a key role in early edema formation after ICH, where thrombin is considered to be responsible for early edema formation [25]. In addition, the possible breakdown of the BBB could lead to the passage of prothrombin from the plasma into the brain parenchyma, where it is converted to thrombin [25]. In the study conducted by Zheng et al., it was noted that thrombin can induce the disruption of BBB function since it activates the phosphorylation of Src kinase, leading to brain microvascular endothelial cell injury, as well as peri-vascular astrocyte injury [26]. It should also be noted that after 2–6 days, Src kinase can promote edema resolution and the recovery of BBB permeability [26]. An additional mechanism by which thrombin can mediate brain injury is through a complement, as it releases nitric oxide, TNF-α, IL-12, and IL-6 [26]. 

Furthermore, a delayed brain edema approximately 3 days post-ICH event is suggested to be caused by red blood cells and mediated by hemoglobin [25]. In experimental models, it was noted that hemoglobin, as well as the products of hemoglobin degradation, including heme and iron, along with carbonic anhydrase, contribute to a brain edema [27].

The role of inflammation was also described in both the process of brain tissue injury and recovery after ICH [27,28]. The inflammation is mediated by cellular (leucocytes, macrophages, T-cells, astrocytes, and microglia) and molecular (prostaglandins, chemokines, cytokines, extracellular proteases, and ROS) components [28]. Aside from the fact that proinflammatory cytokines (IL-1/IL-1β, IL-11, IL-17, IL-23, and TNF-α) have a direct impact on inflammation, they might also contribute to the amplification of the inflammatory response. Contrary to the proinflammatory cytokines, anti-inflammatory cytokines (IL-4, IL-10, IL-33, and TGF-β) contribute to tissue repair [28]. The glycoprotein cytokine IL-6 was shown to have both proinflammatory and anti-inflammatory properties [28].

## 5. Genetic Aspects of Stroke in Pediatric Populations

Genetic causes of stroke in children could be initially overlooked. Early recognition of an underlying inherited condition may facilitate genetic testing for the individual and screening for other family members. Genetic testing is essential for evaluating the patient’s family in order to provide an accurate prognosis and genetic counseling, and may also enable secondary prevention measures for specific conditions [29]. Single-gene disorders have been well studied in relation to stroke [30]. The recent development of molecular genetic methods has greatly facilitated the analysis and diagnosis of this group of disorders. For example, the implementation of Next Generation Sequencing (NGS) panels is a cost-effective approach to diagnose rare genetic diseases that were once excluded from more common causes of pediatric stroke [31].

Using PubMed and Medline, a systematic review of the literature from January 1995 to December 2021 was performed to identify studies addressing the role of genetics in pediatric stroke, as well as studies presenting actual and possible novel stroke therapeutics. The combination of following keywords was used for the primary search: (1) “mutation”, “gene”, “genetic”, ”monogenic”, “hereditary”, “polymorphism”, “genetic susceptibility”, or “genetic risk factors”; (2) “stroke”, “AIS”, “thrombosis”, “ischemic”, or “cerebral infarct”; (3) “neonatal”, “neonates”, “perinatal”, “pediatric”, “childhood”, or “children”; and (4) “therapy”, “treatment”, or “drug”. The publication language was limited to English. Additionally, selected references in the found publications were searched, and particular genes, diseases, and treatments, related to the topic were further investigated. This review includes information from a total number of 167 randomized clinical trials, observational studies, guideline statements, and review articles. The 26 studies describing genetic disorders in which stroke occurs in adulthood have been rejected from the review.

In the following text and Table 1, the most significant single gene disorders associated with pediatric stroke will be presented. Specific therapeutic options will be mentioned whenever they exist. Chapters are organized by mode of inheritance.

### 5.1. Autosomal Dominant Inheritance

Cathepsin A-related arteriopathy with strokes and leukoencephalopathy (CARASAL) is an extremely rare, and very recently described, condition associated with heterozygous mutations in the *CTSA* gene. The first reported patients had persistent hypertension and severe leukoencephalopathy, often accompanied by ischemic or hemorrhagic stroke. During the disease progression, cognitive decline was also observed and, in a single patient from UK, major brainstem features were disturbed. So far, only 19 patients have been reported, with the majority from two distantly related Dutch families, and all patients were heterozygous R325C mutation carriers [32]. After those initial reports, an increase in the CARASAL frequency is expected due to advancing imaging techniques, genetic testing availability, and further investigation of the phenotypic spectrum. To date, there is no evidence that antithrombotic medication, anticoagulation, or thrombolysis is indicated, and no other treatments are available.

Autosomal dominant familial porencephaly, caused by mutations in the *COL4A1* gene, is clinically presented with a spectrum of neurological symptoms, such as seizures, dystonia, hemiparesis, intellectual disability, and migraine, but the severity and age of onset may vary within the same family. Congenital cataracts are frequently observed, as well as pediatric stroke, with serious, life-threatening complications occurring in infancy [33]. Besides the typical cavities filled with cerebrospinal fluid as a result of prenatal and perinatal parenchymal hemorrhage, imaging techniques have been used to discover lacunar infarct and microhemorrhages combined with leukoencephalopathy and calcifications. Porencephaly treatment options, beside physiotherapy, target different neurological symptoms. Antiepileptic drugs are applied for seizure control and surgical intervention is suggested if antiepileptics are ineffective. Additionally, if arterial thrombosis and intraparenchymal hemorrhage or hydrocephalus develops, it is important to identify the source and hemispherectomy, hemispherotomy, and shunts may be included in some cases [34].

Pontine autosomal dominant microangiopathy and leukoencephalopathy (PADMAL) is a rare hereditary subtype of cerebral small vessel disease, caused by mutations in the 3′ untranslated region (UTR) of the *COL4A1* (collagen type IV alpha 1 chain) gene [35]. Several different *COL4A1* noncoding substitutions have been detected in PADMAL, such as the originally reported c.*31G > T, c.*32G > T, and the more recently described c.*34G > T, c.*35C > A. In addition, c.*32G > A is observed in patients with multi-infarct dementia of Swedish type, with most patients having experienced onset after 30 years of age; however, childhood onset is also possible. The proposed mechanism in stroke, due to the above-mentioned mutations, is that *COLT4A1* upregulation in the basement membrane affects the frugality of the vessel walls [36]. 

Cerebral autosomal dominant arteriopathy with subcortical infarcts and leukoencephalopathy (CADASIL) is the most common genetic cause of stroke, induced by inherited mutations in the *NOTCH3* gene, and these mutations are most frequently found in patients with a monogenetic cause of small-vessel disease [37,38]. The cerebral microbleeds affect various brain parts (subcortical white matter, brainstem, cerebellum, thalamus, and basal ganglia) in 34–75 percent of CADASIL patients [39]. The disease clinically presents with strokes and transient ischemic events, accompanied by migraines, psychiatric symptoms, and dementia. CADASIL is typically an adult-onset disease, but may be manifested in childhood. It is recommended to consider CADASIL for children presenting with atypical migraine, suggestive white matter lesions on brain NMR, or a family history of recurrent strokes or transient ischemic attack. Several therapeutic recommendations are implicated in CADASIL patients. Although triptans were not initially recommended for patients with cerebrovascular diseases, a reduction in migraine attacks has been reported in half of CADASIL patients using triptans, without therapy complications. Regarding cognitive impairment, neuropsychological evaluation and support should be applied, and psychiatric treatment is indicated according to the guidelines for cerebrovascular disorders. It is not recommended to prescribe anticoagulant therapy for stroke prophylaxis because of the increased risk of microbleeds in CADASIL, although it may be option if another strong indication is present (e.g., atrial fibrillation, pulmonary embolus) [40].

*ACTA2*-related vasculopathy is an autosomal dominant cerebrovascular disease, recently discovered in patients with a severe phenotype of multisystemic smooth muscle dysfunction (MSMD) syndrome [41]. In the pediatric population, patients with various missense mutations in the *ACTA2* gene affecting the same residue in protein alpha-2 actin (Arg179His, Arg179Cys, Arg179Ser, and Arg179Leu), had a poor prognosis and increased infancy death risk. Numerous features, such as aortic and cerebrovascular disease, mydriasis, underactive bladder and intestine, pulmonary hypertension, and brain white matter anomalies were associated with this syndrome [42]. According to published data, almost 80% of patients had proximal internal carotid artery stenosis and neurological manifestations were reported, including developmental delay and learning difficulties (19% and 7%, respectively), seizures (18%), but also hemiparesis (16%) and spasticity of the lower (6%) and upper (3%) extremities [43,44]. Abnormal brain development, a noticeable characteristic in Arg179Cys mutation carriers, highlights the role of alpha-2 actin these processes [45]. Due to a high risk of perioperative stroke, it is strongly recommended to consider bypass procedures before a reduction of cerebrovascular reserve capacity occurs in patients with *ACTA2* mutations [46].

Marfan syndrome is an autosomal dominant disorder that affects the connective tissue in different organ systems. Mutations in the gene fibrillin-1 (*FBN-1*) disrupt elastic fibers in arteries and, subsequently, increase the risk of arterial dissection (vertebral, carotid, and aorta) [47]. It is estimated that cerebral and spinal ischemia affect up to 20% of Marfan syndrome patients. Dissection of cerebral arteries in patients with Marfan syndrome is usually not related to aortic disease, and Marfan syndrome is included as a differential diagnosis of spontaneous cerebral artery dissection by several guidelines [48]. There is no cure for Marfan syndrome. However, personalized treatment can relieve symptoms and minimize, or prevent, possible complications.

Ehlers-Danlos Syndrome (EDS) type IV encompasses between 5% and 10% of all EDS patients. This autosomal dominant disorder is caused by mutations in the COL3A1 gene (type III collagen). It is mainly associated with vascular complications, including aneurysms, carotid-cavernous fistulae, and cervical artery dissection [49]. Type III collagen is abundant in arterial walls, as well as the walls of the digestive tract, so mutations in COL3A1 cause fragility in these tissues and their predisposition to spontaneous rupture [50]. Additionally, intracranial hemorrhages are identified in about 4% of cases. The rupture of a previously identified intracranial aneurysm was the cause of bleeding in a half of those patients. Therefore, EDS type IV should be considered after ischemic stroke in children. Although EDS is not yet curable, individually tailored treatments may be helpful for different symptoms.

Hutchinson-Gilford Progeria Syndrome (HGPS), a rare, fatal condition that begins in early childhood, is characterized by segmental, premature aging. HGPS is caused by sporadic heterozygous mutations in the *LMNA* gene, which codes for progerin, an inner nuclear membrane protein lamin A. The mutated progerin production leads to an aberrant nuclear shape and, subsequently, disturbs the regulation of numerous genes. Vascular abnormalities in the brain are common in HGPS patients due to the expression of progerin in cells that are essential for vascular structure and function [51]. Additionally, AIS was detected in 60% of progeria patients between the ages of 5 and 10 years, even without clinical manifestations. Regarding distribution, large-vessel territorial infarctions predominantly affect the middle cerebral artery and watershed regions, but ischemic injury of white matter within the cerebral hemisphere and lacunar infarcts could also be seen as a result of chronic hypoperfusion [51,52]. Although a cure for progeria has not yet been developed, physical therapy may be helpful for stiff joints. Pilot data by Ullrich et al. showed that treatment with the oral farnesyltransferase inhibitor lonafarnib may alter the progression of cerebrovascular disease within a relatively short period of time [53].

Von Hippel–Lindau (VHL) disease is a dominantly inherited syndrome caused by germline mutations in the *VHL* tumor suppressor gene. The disease clinically presents with an increased risk of simple visceral cysts development, predominantly in the kidneys and pancreas [54]. The later disease course is accompanied by the development of multiple neoplasms, such as haemangioblastoma (HB) in the CNS, clear cell renal cell carcinoma, phaeochromocytomas, pancreatic islet tumors, and endolymphatic sac tumors [55]. HB are vascular tumors associated with an increased risk for hemorrhagic stroke. Although VHL disease is fully developed in early adulthood, early screening and follow up of neoplastic lesions is highly recommended to begin by the age of two.

Neurofibromatosis type 1 (NF1) is an autosomal dominant disorder associated with the *NF1* gene, which encodes the protein neurofibromin. Clinical presentation frequently includes café-au-lait spots, neurofibromas, optic pathway gliomas, Lisch nodules in the eyes, and skeletal abnormalities [56]. Besides the predisposition to certain types of neoplasms, NF1 may include vascular abnormalities, usually developing early in life. Vascular malformations can affect any organ, but the effect on vessels from the CNS has the most prominent effects on morbidity and mortality [57,58]. An association with vascular abnormalities including moyamoya arteriopathy, cerebral aneurysms, and stenotic or ectatic cerebral vessels increases the risk of ischemic and hemorrhagic stroke in affected individuals [59].

Neurofibromatosis type 2 (NF2) is a tumor-prone disorder caused by the heterozygotic inactivation of the *NF2* tumor suppressor gene, which encodes for the protein merlin. Truncating *NF2* mutations (e.g., nonsense and frameshift mutations) usually lead to a severe phenotype, while missense mutations often lead to a milder clinical picture, and splicing *NF2* mutations could be associated with various phenotypes [60]. An NF2 phenotype is presented in children as multiple spinal and intracranial tumors, rapid hearing loss, and cranial nerve palsies (cranial nerves III to VIII) [61]. In pediatric NF2 patients, several cases of ischemic stroke of the brain stem and of cerebral aneurysms have been reported in the literature [60].

Lifetime monitoring begins as soon as the diagnosis of NF1 or NF2 is suspected. Due to the complexity of the disease, the management of children with NF requires communication among the various specialties, and the standard therapy is still surgical. The conception of innovative molecular-targeted drugs is based on a better understanding of the molecular biology of the *NF1* and *NF2* genes, as well as the molecular pathogenesis of NF1 and NF2. The development of mouse models of NF1-associated malignant disease plays important role in this process [62,63].

### 5.2. Autosomal Recessive Inheritance

Sickle cell disease (SCD) is one of the most common causes of pediatric stroke. The children with the highest risk possess homozygosity of β^S^ alleles in the gene encoding the β chain of hemoglobin, which is the most severe and prevalent genetic type. SCD brain vasculopathy can give rise to both manifested stroke and “silent” cerebral infarcts, disturbing neurological and cognitive function [64,65]. It is assumed that the stroke risk in SCD patients depends on many genes, both within and outside of the *HBB* gene locus, so that pathogenesis depends on a combination of the β^S^ mutation of the gene *HBB*, some genetic modifiers, and environmental factors. GWAS and exome study, conducted by Flanegan et al., included an initial cohort of 677 children and a validation cohort of 288 children, and indicated that two mutations in the genes *GOLGB1* (Y1212C) and *ENPP1* (K173Q) were considerably associated with a decreased risk for stroke [66]. The mutation in the *GOLGB1* Y1212C gene has also been found to be protective against silent infarcts and an abnormal transcranial Doppler (TCD) [66,67]. Opposite to these findings, Belisario et al., in their longitudinal study, revealed that the variant *ENPP1* K173Q was associated with an increased risk of stroke and pointed to an increased incidence of high-risk TCD [68]. The standard care for SCD patients with an acute neurologic deficit is urgent blood transfusion therapy and, if possible, exchange transfusion. For the prevention of stroke recurrence after a regular transfusion, if an HLA-matched sibling donor is available, the haematopoetic stem cell transplantation (HSCT) is recommended, regardless of age. If a donor is not available, a regular exchange transfusion should be implemented in order to keep the maximum HbS level below 30%. If regular blood transfusion is not applicable, therapy with hydroxyurea is recommended as a better choice than no therapy at all [69]. Very recently, published results of a phase 1-2 study showed the promising biological and clinical efficacy of SCD gene therapy with the use of LentiGlobin [70].

Grange syndrome is determined as a severe, early-onset vascular occlusive disease that may be accompanied with brachydactyly, syndactyly, bone fragility, and learning disabilities. This condition is caused by biallelic loss-of-function mutations in the *YY1AP1* gene, so it is inherited in an autosomal recessive manner [71]. For example, Saida et al. revealed a novel, homozygous, pathogenic, out-of-frame deletion in the *YY1AP1* gene (c.1169del: p.Lys390Argfs*12) in a patient who had an intracerebral hemorrhagic stroke, but no brain artery anomalies or bone fragility [72].

Thrombotic thrombocytopenic purpura (TTP) results from a severe deficiency of the specific von Willebrand factor (VWF)-cleaving protease named ADAMTS13 (a disintegrin and metalloprotease with thrombospondin type 1 repeats, member 13). This rare thrombotic microangiopathy is presented as microangiopathic hemolytic anemia, severe thrombocytopenia, and ischemic organ injury due to microvascular thrombi rich in platelets. Upshaw–Schulman syndrome, or hereditary thrombotic thrombocytopenic purpura (HTTP), is a congenital TTP characterized by a persistent and severe ADAMTS13 deficiency (<10%) caused by homozygous or compound heterozygous pathogenic mutations in the *ADAMTS13* gene [73]. Stroke occurs in 25–31% of patients with HTTP, with the median age of 19 years and 22% of strokes occurring before the age of 10 years [74].

ADAMTS13 thrombotic microagiopathy occurs due to the production of a nonfunctional ADAMTS13 protein caused by compound heterozygous or homozygous mutations of the *ADAMTS13* gene. Members of the ADAMTS gene family, primarily *ADAMTS13*, are extracellular matrix components which, in combination with misbalanced coagulation signals, may have an important role in the etiology of pediatric stroke after postnatal vascular injuries. Recently, Witten and colleagues have reported results of a family-based genome wide association study (GWAS) for pediatric stroke, and suggested a possible role of *ADAMTS12* in pediatric stroke [75]. The ADAMTS13 activity and levels are good predictors of the occurrence and prognosis of ischemic stroke. In addition, animal studies on ADAMTS13 in the treatment of AIS are delivering promising results, especially in the model of conformational activation; as such, ADAMTS13 may become a new therapeutic agent for ischemic stroke [76].

Cerebral autosomal recessive arteriopathy with subcortical infarcts and leukoencephalopathy (CARASIL) is a genetic disorder characterized by mutations in the *HTRA1* gene. It is a very rare autosomal recessive form of ischemic, non-hypertensive, cerebral small vessel disease. Patients usually develop several phenotypes in their early lifetime (teenage years or 20–30 years old) [77]. Typical clinical features of CARASIL are alopecia, gait disturbance, and dementia. Severe changes in white matter occur as a consequence of recurrent cerebral infarcts and could cause gait disturbance and dementia. Infarcts or bleedings due to the cerebral small vessel disease can lead to significant neurological deficits and even cause death [78]. Due to insufficient evidence, antiplatelet therapy is not recommended in CARASIL, even in the presence of cerebrovascular events.

Deficiency of adenosine deaminase 2 (DADA-2) is a rare, autosomal recessive, autoinflammatory disease. This disease occurs as a result of compound heterozygous or biallelic homozygous mutations in the *CECR1* (Cat Eye Syndrome Chromosome Region 1) gene. Deficiency of ADA-2 can lead to vasculopathy and inflammation through the macrophages and the polarization of monocytes onto proinflammatory cells, resulting in damage to the endothelial integrity. Vasculopathy, associated with hemorrhagic and ischemic strokes in ADA2 deficiency, has an early-onset as well as clinical and histopathological characteristics of polyarteritis nodosa (PAN) [79]. DADA2 is predominately expressed in childhood. About a quarter (24%) of cases were reported before the first year of life and 77% before the age of 10 [80]. If the genetic tests do not confirm the diagnosis in the patient, additional functional assays to measure ADA2 activity should be performed [81]. Early diagnosis of the disease and forehand treatment are crucial for the prevention of severe complications. Treatment of DADA-2 is based on TNF-inhibition in order to suppress inflammation and prevent vascular occurrence. For the patients with hematological disease and immunodeficiency who do not respond to TNF-inhibitors, hematopoietic stem cell transplantation (HSCT) may be an option. Possible novel therapies include recombinant ADA2 protein or gene therapy [82].

Pseudoxanthoma elasticum (PXE) is an autosomal recessive metabolic disease. Mutations in the *ABCC6* gene cause a deficiency of a functional ABCC6 protein that provokes slow, progressive, abnormal mineralization and fragmentation of the elastic fibres in affected tissues. Clinical features include typical skin lesions (small, yellowish bumps), ocular angioid streaks, and multisystem vasculopathy, as the mineralization of the soft connective tissue is the most conspicuous in the skin, eyes, and the arterial blood vessels [31,83]. Reported vascular manifestations of PHE encompass stenosis of the aorta and medium-sized arteries, such as the radial and carotid arteries, transient ischemic attacks, stroke, and aneurysms [84]. PXE may present in childhood with isolated multisystem vasculopathy and early-onset stroke [31]. Regarding therapy of PXE, promising molecular approaches include the correction of nonsense ABCC6 mutation read-through of translation by a non-aminoglycoside nonsense mutation suppressor molecule PTC124 and corrections of the cellular localization of the mutant protein by the chaperon-assisted allele-specific therapy with 4-phenylbutyrate (4-PBA). One of the promising cell-based treatments, which is still under investigation, is the transplantation of allogenic mesenchymal stem cells (MSCs), which has demonstrated the homing of cells to the liver and their ability to contribute to liver regeneration. Additionally, liver transplantation or a partial lobe replacement should be considered in order to protect ABCC6 activity [85].

Ehlers-Danlos syndrome (EDS) type VIA (the kyphoscoliotic type) is a rare autosomal recessive disorder which arises due to a mutation in the *PLOD1* gene. The syndrome is characterized by connective tissue dysplasia, which leads to hypotonia, malignant kyphoscoliosis, hyperlaxity, hyper-elasticity, and skin fragility. In several cases of a prenatal brain stroke, a biallelic duplication of exons 10 and 16 in the *PLOD1* gene was found [86].

Acardi–Goutières Syndrome (AGS) is a rare, inherited, multisystem disorder associated with a vasculopathy leading to ischemic stroke. Specific mutations in the RNASEH2A, RNASEH2B, RNASEH2C, SAMHD1, or TREX1 genes, involved in DNA replication and repair, are responsible for AGS. The age at presentation and the clinical phenotype are dependent on the underlying gene defect. Patients with a TREX1 mutation typically have symptoms at birth, and those with RNASEH2B mutations develop symptoms within a few months of birth [28]. AGS is most frequently inherited in an autosomal recessive manner. This syndrome can also occur as a consequence of specific pathogenic variants in ADAR or TREX1, which arise de novo or are inherited in an autosomal dominant manner, with various heterozygous autosomal dominant pathogenic variants in IFIH1 [87]. There is no current cure for AGS, only symptomatic management can be done at this time.

### 5.3. X-Linked Inheritance

Menkes disease is an X-linked recessive, early-onset (6 to 8 weeks of age), neurodegenerative disease leading to an early death, often before three years of age. This disease occurs as a consequence of a copper metabolism disorder that manifests as seizures, hypotonia, failure to thrive, hair abnormalities, and connective tissue malformations. Other features associated with the clinical phenotype include abnormalities in the intracranial vasculature, such as increased intracranial vessel tortuosity, variable abnormalities of white matter signal, transient temporal lobe changes, Purkinje cell degeneration in the cerebellum, cerebral and cerebellar atrophy, basal ganglia malformations, and subdural collections. Menkes disease arises as a consequence of loss-of-function mutations in the *ATP7A* gene, which encodes ATPase Copper Transporting Alpha, causing the impaired absorption and cellular metabolism of copper [88,89]. Early treatment of Menkes disease is essential, starting within 28 days of birth. Copper supplementation may be effective in cases where the mutated protein retains some residual function [90]. In some patients, the application of a freeze-dried copper histidinate (CuHis) showed increasing amount of copper in the blood and improved neurodevelopmental outcomes. One of the developing treatments with promising results in Menkes disease therapy is an adeno-associated viral gene therapy in combination with subcutaneous CuHis injections [91].

Fabry disease (FD) is a rare, inherited, lysosomal storage disease caused by mutations in the *GLA* gene, which manifests as a lack, or significant deficiency, of the lysosomal enzyme α-galactosidase A (α-GAL A), leading to the accumulation of globotriaosylceramide (GL-3) within lysosomes in different cell types, including endothelial cells, podocytes, and cardiomyocytes [92]. Although the most often affected organs in FD are the heart and kidneys, cerebrovascular complications are also seen [93]. Stroke is considered to be a manifestation of end-stage Fabry disease. However, in registries, strokes occurring as early as childhood have been recognized. Consistent with a 12-fold increased risk of transient ischemic attack and stroke in patients with FD, the strokes are usually ischemic [94]. The treatment of Fabry disease is based on enzyme replacement therapy (ERT) (agalsidase alfa and agalsidase beta) and the chaperone migalastat. Exploration of new therapeutic possibilities include new forms of ERT, substrate reduction therapy, mRNA therapy, and gene therapy [95].

### 5.4. Mitochondrial Inheritance

Mitochondrial myopathy, encephalopathy, lactic acidosis, and stroke (MELAS) syndrome is considered to be most frequent mitochondrial disease. The MELAS inheritance is associated with the mitochondrial genome. It is, in its course, progressive and affects multiple systems, leading to a neurological pathology, disability, and, ultimately, death. Although symptoms of MELAS usually occur between the ages of two and fifteen, cases of delayed onset have also been reported. Therefore, a timely diagnosis, along with genetic counseling, might be of benefit in improving the prognosis of these patients [96]. MELAS might be presented with an acute metabolic stroke. In about four out of five patients, MELAS syndrome is connected with a point mutation in the *MTTL1* gene that encodes tRNA-Leu (m.3243A>G). Other individuals that are diagnosed with a MELAS have other variants of *MTTL1* or mutations in some other mitochondrial genes [97]. Considering potential therapeutic options, the administration of L-arginine and L-citrulline could elevate nitric oxide (NO) production, thus having favorable benefits for patient care with MELAS [98,99].

### 5.5. Somatic Mutations

Struge–Weber syndrome (SWS) is a congenital, neurocutaneous disorder with vascular malformations. This disease is characterized by an abnormal formation of tiny blood vessels in the facial skin (port-wine stain), along with malformations of veins and capillaries in the brain and eyes. Vascular malformations may also be seen in other locations, such as the buccal cavity or the respiratory tract. A few cases of SWS associated with venous thrombosis and intracranial hemorrhage have been reported to date. This congenital disease is predominately sporadic, even though some familial cases have been described [100,101]. A somatic mosaic mutation (c.548G>A; p.R183Q) of the *GNAQ* gene is detected in the brain or skin of more than 80% of patients from different populations. In both patients with classic SWS, and patients who only have a port-wine stain, this *GNAQ* gene mutation disrupts the activity of the encoded guanosine triphosphatase [102]. Serious neurological symptoms with variable clinical courses in patients with Sturge–Weber syndrome include transient episodes of hemiparesis that resemble ischemic strokes. In patients who have these “stroke-like episodes”, changes typical for arterial stroke were very rarely found on the brain MRI. [103]. Standard treatments for SWS patients include laser therapy for the birthmark, eye drops or surgery for glaucoma control, and anticonvulsants. Optionally, anticonvulsant or low-dose aspirin treatment, or both, before the onset of seizures may be applied. To the patients whose seizures are medically refractory, surgical resection may be offered [104].

### 5.6. Multifactorial Disorders

Moyamoya syndrome (MMS), or Moyamoya disease (MMD), is presented as the progressive stenosis of the distal intracranial internal carotid artery (ICA) and, less commonly, as the progressive stenosis of the proximal anterior cerebral artery (ACA), middle cerebral artery (MCA), basilar artery, and posterior cerebral artery. Moyamoya disease commonly appears in children, in all ethnic groups, with a peak incidence at 5 years of age [105]. Pediatric MMD is usually manifested by cerebral ischemia (80%) rather than hemorrhage (20%) [106]. Recurrent transient ischemic attacks or ischemic strokes are frequent in children. Without therapy, 50% to 60% of patients with MMD recurrent strokes result in the gradual deterioration of neurologic and cognitive function [107].

The strong ethnic bias (in East Asians), and 15% of familial cases, strongly implicate a genetic basis of MMS. Several HLA alleles, including HLA-B35, HLA-B51, HLADRB1*04:05, DQB1*05:02, and *04:01, have been associated with MMD in East Asians through genetic linkage analysis. Moyamoya disease was also described in association with some genetic syndromes, including trisomy 21, Turner syndrome, sickle cell disease, and neurofibromatosis type 1 (NF1). In Japanese and Korean populations, a polymorphism in the *RNF213* gene (p.R4810K) was associated with MMD. Regarding the prevalence of MMS as 6/100 000 and a 1% carrier rate of p.R4810K in the Japanese population, it is obvious that some additional factors contribute to the expression of Moyamoya disease in carriers. The same polymorphism was noticed in a small number of East Asian patients who had some of the genetic syndromes with a predisposition to MMD, including NF1 and trisomy 21. These findings suggest that *RNF213* may act as an additional modifier. Evidence from *RNF213* knockouts indicate that a loss-of-function mutation could lead to brittle blood vessels, enhanced susceptibility to hemodynamic stress, and other vascular insults. Additional proposed mechanisms include a proinflammation, resulting in endothelial cell dysfunction and the proliferation of smooth muscle cells with vascular stenosis [108].

Therapy for MMD is still based on the supportive role of medications, such as aspirin and calcium channel blockers, in the absence of a definitive medical treatment to reverse or stabilize the course of the disease. Additionally, there is no standardized surgical treatment of MMD in children. In order to establish revascularization, different surgical procedures have been used. They all aimed to prevent further ischemic injury by using external carotid circulation as a donor supply in order to increase collateral flow [106].

Surgical revascularization procedures are particularly used for patients with cognitive decline or recurrent or progressive symptoms. Some procedures, including direct anastomosis, which is most commonly a superficial temporal artery to MCA anastomosis, are difficult to perform in children due to the small size of scalp donor vessels or MCA recipient vessels. In order to overcame this problem, some new procedures, including encephaloduroarteriosynangiosis and encephalomyoarteriosynangiosis, have been developed for indirect bypass [105,109]. A procedure with promising results in promoting neovascularization is the pial synangiosis (a modification of the encephaloduroarteriosynangiosis), which is based on the transposition and fixation of the superficial temporal artery to the brain surface. In a review of 143 children with moyamoya disease, it was demonstrated that a treatment with a pial synangiosis decreased their stroke frequency after surgery. However, surgical treatment in MMD patients can lead to complications such as postoperative ischemic stroke, spontaneous or traumatic subdural hematoma, intracranial hemorrhage, and infection [105,107].

PHACE is a syndrome with unknown pathogenesis and sporadic occurrence. This syndrome was named after an acronym of the leading signs: posterior fossa anomalies, hemangioma, arterial anomalies, cardiac anomalies, and eye abnormalities. Without reported cases of familial recurrence or occurrences of PHACE in the offspring of adult women with PHACE, there is no evidence about heredity in this disease; however, in the woman who have given birth to children with PHACE syndrome, a higher incidence of pre-eclampsia and placenta previa during pregnancy was reported [110]. Many authors suggested that PHACE arose as a consequence of a postzygotic somatic mutation occurring between approximately VI and IX gestational weeks. De novo genetic variants, copy number variants, epigenetic mechanisms, and possible in utero environmental factors or hypoxic events may also be the cause of PHACE syndrome. Array-CGH analysis did not detect any large (>130 kb), rare copy number variant (CNV) regions common for the multiple patient with PHACE. Large, rare duplications/deletions at chromosomal regions 1q32.1, 3q26, 3p11.1, 10q24.32, 12q24.13, 17q11.2, and 18p11.31 were reported in single individuals. In some of these regions, mapped genes that play roles in the development, angiogenesis, and matricellular signaling, including *PIK3CA* (3q26), *EPHA3* (3p11.1), and *EMILIN2* (18p11.31), can be found [111]. Whole exome sequencing identified variants in the *RNF213* gene in several patients who presented with PHACE in early childhood and may be at risk for progression of moyamoya vasculopathy over time, therefore providing evidence that genetic variants are associated with progressive arteriopathy, including moyamoya vasculopathy, in PHACE as well [111].

Treatment of PHACE syndrome is multifaceted. Oral propranolol can be applied to the patients with large and problematic haemangiomas, with special caution in those with concurrent anomalies of cerebral arteries, when beta-blockers can cause a predisposition to ischemic stroke. Specialist-led multidisciplinary follow-up, including a pediatric neurologist, cardiologist, ophthalmologist, endocrinologist, speech and language therapist, and orthodontist, is essential in children with PHACE syndrome. PHACE syndrome is a life-long disorder and, therefore, it is very important that the psychological health of children with PHACE syndrome is well attended to as well [112].

## 6. Genetic Risk Factors in Pediatric Stroke

**Figure 1:**
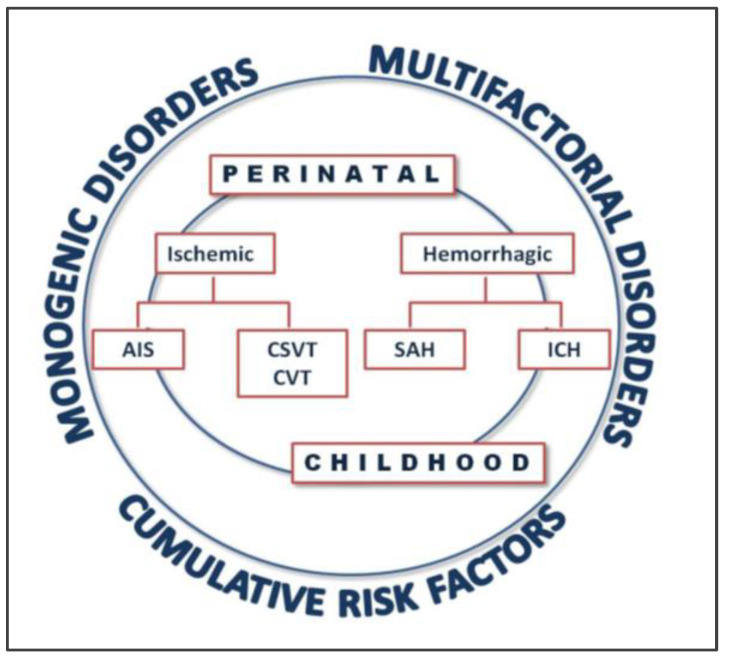
Schematic presentation of pediatric stroke causes. AIS, arterial ischemic stroke; CSVT, cerebral sinovenous thrombosis; CVT, cortical vein thrombosis; SAH, subarachnoid hemorrhage; ICH, intracerebral hemorrhage.

As it was stressed earlier, the etiology of stroke in children is highly complex and results from the convergence of multiple factors (Figure 1). Additionally, the leading causes of perinatal and childhood stroke are different and very much variable, although a genetic component can reasonably be expected to be a predominant contributing factor in pediatric stroke, especially due to do lack of other risk factors (common in adult stroke) and the young age of the patients. Besides the above-mentioned monogenic disorders that may present with stroke as a first symptom, numerous studies indicate that genetic polymorphisms may contribute to the risk of pediatric and perinatal stroke.

The main issue in establishing the impact that certain genetic risk factors have on stroke in children is the fact that, fortunately, it is a rare condition. Studies are not very common and usually encompass a small number of patients. This state-of-the-art review is not going into detail of the reliable interpretations and constructive recommendations that may be useful in clinical settings.

The most frequently studied genetic risk factors are several common polymorphisms in genes associated with thrombophilia, which code for proteins that are part of the coagulation cascade, fibrolysis, homocystein metabolism, lipid metabolism, or platelets (Table 2).

The methylenetetrahydrofolate reductase (*MTHFR*) C677T polymorphism affects protein thermostability and decreases enzymatic activity, resulting in elevated homocysteine levels and, subsequently, an increased risk of cerebrovascular diseases, including stroke [148]. Correlation of *MTHFR* C677T polymorphism and pediatric stroke has been observed in different populations [113,114,115,116,117,118,119,120,121,122], although some studies did not report an association [123]. Two meta-analyses that included 11 and 15 case-control studies (777/822 CAIS and 1715/1552 matching controls, respectively) confirmed that the above-mentioned polymorphism increases the risk of AIS during childhood [124,125]. 

For another polymorphism in the same gene, A1298C, it has been shown that it mildly reduces MTHFR activity [149]. A suggested association of the polymorphism and pediatric stroke is investigated in several studies with conflicting results [115,116,119,121,122,126,127,128]. All findings are included in a recent meta-analysis, with the conclusion that the *MTHFR* A1298C polymorphism is not related to CAIS [129].

Among different risk factors that may influence the risk of pediatric and perinatal stroke, polymorphisms in genes encoding hemostatic regulatory proteins are important targets. One of the most frequently investigated genetic risk factor among coagulation factors is the polymorphism in the *factor V* gene, named *FV* Leiden. This amino acid change activates protein C resistance, and subsequently leads to a susceptibility to thrombosis [150]. *FV* Leiden is undeniably associated with an increased risk of pediatric stroke in a significant number of studies, and is further confirmed in a meta-analysis [124]. Additionally, an association is confirmed in different subtypes of pediatric ischemic stroke, as well as part of several haplotypes [130].

One of the prothrombotic risk factors, *factor II* (prothrombin) G20210A polymorphism, is associated with an increased prothrombin level and the reduced conversion of fibrinogen to fibrin, which may lead to hypercoagulability [151]. A recent meta-analysis included 921 pediatric patients with AIS and 2354 healthy newborns and children from different populations, with predominant population being Caucasians [131]. The majority of the 14 analyzed studies addressing the effects of this polymorphism on AIS in children and neonates suggested an association; however, due to the small number of patients and controls in most studies, some results were inconclusive or even contradictory [113,115,116,117,118,120,132,133,134,135,136,137,138,139]. Results from the meta-analysis confirmed that A allele carriers are statistically more common in pediatric patients with AIS than in controls. Additionally, six analyzed studies included cohorts of perinatal ischemic stroke patients and the same trend is observed in perinatal AIS, but without statistical significance [131]. Previous systematic analyses also showed an association of the *FII* G20210A polymorphism and pediatric AIS [124,140].

A systematic analysis included four family-based and case-control studies of the *FXIII* polymorphism V34L in CAIS [115,128,133,141]. Although a slightly increased risk of childhood AIS was identified in some studies [128], no significant differences in allele distribution or genotype were reported, and a recent meta-analysis of 358 children with stroke and 451 controls from different populations confirmed no association between the *FXIII* polymorphism 34L allele and CAIS [142].

A meta-analysis of the in/del polymorphism 4G/5G in the promoter region of the *SERPINE1* gene, coding for the plasminogen activator inhibitor (PAI-1), included 8 studies [115,118,128,134,143,144,145,146]. In total, 600 children with stroke and 2152 controls from mostly European, Turkish, and Thai populations were analyzed, and a correlation between the 4G allele and CAIS was not observed [142]. The data of the PAI-1 polymorphism and susceptibility to ischemic stroke in adults was inconsistent, but a recent meta-analysis based on 44 studies suggested an increased risk, especially in Asian and Mixed populations [152]. These findings confirm the difference between genetic susceptibility to stroke in children and adults.

Platelet glycoprotein receptor polymorphisms, named human platelet antigens (HPAs), modulate receptor density and, subsequently, platelet function and thrombus formation [153]. The association of HPAs with pediatric and perinatal AIS is poorly investigated and, in most studies, only HPA-1 antigen variants on glycoprotein IIIa are included. An increasing mild risk was observed among patients with ischemic pediatric stroke, especially with CSVT, a carrier of at least one HPA-1b allele, although the results are inconsistent [115,118,122,128,130]. Additional antigens, HPA-2, HPA-3, and HPA-5, are analyzed in just a few studies and interesting findings suggest a lower risk for both pediatric and perinatal AIS, but an increased risk for CSVT among carriers of the HPA-3b allele [128,130].

Apolipoprotein E, a component of cerebrospinal fluid lipoproteins, is coded by the *APOE* gene. Two polymorphisms (rs429358 and rs7412) create 3 alleles (e2–4) that encode three protein isoforms with different binding capacities to other proteins and phospholipids [154]. Although *APOE* polymorphisms are investigated in adult patients with ischemic stroke, an association in children is explored in only a few studies, and the results did not confirm an association [116,128,147].

Another important aspect in studying the genetic risk factors of pediatric AIS is to establish their involvement in stroke recurrence. Although perinatal stroke is a frequent subtype of stroke in children, recurrence is not reported to be very common and an association with thrombophilia genetic risk factors, such as *FV*, *FII*, or *MTHFR* (C677T) polymorphisms was not observed [155,156]. A recent, large, international study was focused on the effects of thrombophilia risk factors on stroke recurrence in children [157]. The main goal of the study was to stratify patients according to the risk of a recurrent stroke in order to intensify secondary prevention. The study included a remarkable number of 894 patients with pediatric first AIS and, among those patients, 160 cases of recurrent stroke. The *FV* Leiden was associated with the second stroke and combined defects in every fourth patient, but the *FII* 20210G>A polymorphism was not significantly associated with recurrent stroke. The authors pointed to a possible cumulative effect of prothrombotic susceptibility factors on the increased risk of recurrence. A cumulative pattern is suggested, not just for recurrent stroke, but also for children’s susceptibility to AIS [128,130].

## 7. Up-and-Coming Therapeutic Modalities and Approaches

Recent steps forward in our understanding of the disorders underlying strokes have given us a next generation of therapeutics and therapeutic targets by which to improve stroke survival, protect or rebuild neuronal connections in the brain, and enhance neural function.

The heterogeneous etiology and variable clinical presentations of stroke in a pediatric population complicates the timely diagnosis and adequate treatment of pediatric stroke, subsequently causing significant morbidity and mortality [158,159]. In children, acute ischemic stroke (AIS) is associated with recurrent strokes, lifelong disability, and decreased quality of life [159]. 

Early recognition of AIS is crucial, together with adequate diagnostic procedures, including neurologic examination and neuroimaging (preferably Magnetic Resonance Imaging—MRI) [160]. The primary treatment is based on the supportive therapy. Current recommendations are to apply thrombolytic agents or clot-dissolving drugs, such as alteplase (an intravenous recombinant tissue plasminogen activator—rTPA) in children with acute ischemic stroke recognized within the recommended 4.5 h therapeutic window [158,161,162]. As the administration of a thrombolytic therapy may increase the risk of intracranial hemorrhage in children, a pediatric hematologist, in addition to a neurologist, should be included in the assessment of therapeutic possibilities. Recent research, based predominately on the case reports or the case series, suggest that endovascular treatment in children seems to be a feasible and safe treatment with favourable outcomes [163,164,165,166]. Thrombectomy, in order to establish revascularization, has been performed in cases of a large vessel occlusion in children, and has had a good neurological outcome [159]. Critical considerations about endovascular thrombectomy in children refer to the risk of vasospasm during the procedure, blood loss, radiation exposure, and contrast administration [167]. In order to prevent complications, both thrombolytic and endovascular treatment should be performed based on the individual case.

Additionally, an early recognition of stroke and stroke risk in the pediatric population might influence the improvement of outcomes and quality of life of the patient, as well as their families, by potentially advancing the neuroprotective, thrombolytic, and antithrombotic treatments, as well as rehabilitation strategies early after the onset of stroke [168,169]. 

Neural repair can be referred to CNS structural or functional restoration after an injury, including stroke. Numerous categories of repair-based therapies are currently being studied. Such types of treatments are distinct from prevention-based treatments, as well as those that aim to reduce acute injury, such as reperfusion or neuroprotection. The treatment time window for repair-based therapies may last days, weeks, or even longer, so a potentially higher number of patients with stroke may be accessed [170]. 

### 7.1. Stem Cells

Stem cells are among the promising potential therapeutic modalities for individuals with ischemic stroke that could help in the reconstruction of neuronal circuits after chronic stroke and extend the therapeutic window of stroke treatment. Stem cell therapies for stroke aim to repair, replace, and enhance the biological function of damaged or dead cells to restore neural integrity [171]. Studies on animal models have pointed out the beneficial effects of stem cells such as embryonic stem cells (ESCs), inducible pluripotent stem cells (iPSCs), mesenchymal stem cell (MSCs), as well as neural stem cells (NSCs) because of the potential for the cell replacement, neuroprotection, endogenous neurogenesis, angiogenesis, and modulation on inflammation and the immune response [172].

A rising amount of evidence during the last two decades has highlighted the possibility of the application of hematopoietic growth factors and different stem cell transplantation in stroke therapy [173]. Clinical trials that would address the reprogramming of human peripheral blood mononuclears to NSCs are difficult to perform due to the high risk of mutation accumulation during the processes, and, subsequently, the possible carcinogenicity [174]. 

Tsai and colleagues presented successful results of their clinical trials, demonstrating granulocyte colony-stimulating factor (GCSF) administration, as well as bone marrow mesenchymal stem cells (MSCs) (CD34^+^) mobilized by GCSF in stroke treatment. The preliminary results of the stereotactic implantation of autologous adipose tissue-derived stem cells (ADSCs) are indicating their safety and improvement of sensorimotor function without carcinogenicity, immune rejection, or ethical concerns [173,175]. Berlet and colleagues point to the fact that there could be synergistic effects of combined stem cell transplantation and rehabilitation therapies that lead to the improved growth, migration, maturation, and neural differentiation within the stroke injury area [176]. At present, a single clinical study is addressing the usage of MSCs in the treatment of neonatal stroke. The phase 1 dose-escalation trial of intratracheal administration of allogeneic umbilical cord-derived MSCs was performed in 9 preterm infants with a risk of for bronchopulmonary dysplasia. Chang et al. did not report any serious adverse event in the trial, and positive results are expected from the current, similar study in preterm neonates with severe intraventricular hemorrhage (ClinicalTrials.gov: NCT02274428) [177,178].

### 7.2. Exosomes

Exosomes can be considered as extracellular particles that are small and non-toxic, and are discharged by a different cell types that are biologically active, particularly from, but not limited to, the epithealial, immunological, and tumorous cells, as well as neurons and the glia. They consist of heterogenous substances that include proteins and lipids, as well as DNA fragments, mRNAs, and miRNAs [179]. The exosomes might have a positive potential in the treatment of neurological diseases, including ischemic post-stroke conditions, due to their efficient crossing of the blood–brain barrier while not provoking an immune response. Furthermore, it was shown that they have an ability to be retained by peripheral organs, and thus, may play a crucial role in cerebral ischemia [179,180]. Different, non-coding RNAs in exosomes can enhance the neural communication and improve the regulation of the development and regeneration of the neuronal cells and myelin synapses, remodeling of blood vessels, inhibition of neuroinflammation, and maintenance of homeostasis of the nervous system. Moreover, exosomes are also suitable carriers of bioactive substances, which can be modified and targeted to the lesion site [181]. Previously, it was stated that miR-124 has proneuronal activities for both the developing and mature brain. Exosomes containing miR-124, through the processes of cortical neurogenesis, promote cortical neural progenitors to achieve neuronal identity and confer recovery after ischemia [182]. The exosomes produced by endothelial progenitor cells (EPCs) contain high amounts of miRNA-126 and miRNA-296, and have an effect of promoting angiogenesis and anti-apoptosis [183]. Xin et al. have demonstrated increased neurogenesis, oligodendrogenesis, and neural plasticity in stroke-injured areas in rats after an intravenous injection of exosomes containing a miRNA-17–92 cluster [184]. Zhang et al. found that human neural stem-cell-derived exosomes produced under hypoxia preconditioning can promote the treatment of a stroke [185]. In the study conducted by Yang and colleagues, the exosomes were used to deliver the circular RNA to the ischemic zone. Neuronal cells were particularly targeted through the expression of the RVG (rabies virus glycoprotein) peptide on the membranes of the exosomes, and used this RVG-Exo as a cargo delivery system for Circ-SCMHI RNA. An intravenous injection of RVG-CircSCMH1-Exo was shown to have beneficial effects on motor recovery improvement as well as functional recovery in both rodents and non-human primate models. It was suggested that RVG-CircSCMH1-Exo could have a wider therapeutic potential window compared with current therapies, since they might be administered 24 h after stroke onset [186]. In addition, as exosomes have lipid membranes with a hydrophilic core, which is important for the delivery of soluble drugs, they are suggested as a drug delivery system for the nervous system, which requires efficient and non-toxic drugs [183]. Another possibility is loading genes by exosomes. One example is delivering brain-derived neurotrophic factor (BDNF) to injured tissue and to promote nerve recovery due to the neuroprotective function of BDNF in MCAO-treated mice [187]. These findings may support the idea of introducing certain genes into tissue affected by stroke to support blood vessel regeneration [183].

Considering exosome therapy for stroke, there is one registered clinical trial thus far, NCT03384433, where scientists are investigating the effects of allogenic MSC exosomes enriched with miR-124 on disability improvement in a population with AIS. For this clinical trial, eligible individuals will receive 200 mg of MSC exosomes by stereotaxic administration that were transfected by miR-124 one month after the AIS onset. Twelve months following the administration of the treatment, patients will be evaluated by the modified ranking scale, while adverse treatment-related events will be analyzed as well [179]. Clinical trials NCT04175691 and NCT04230785, through the application of next-generation sequencing in a targeted population, will evaluate the patterns of expression for circular RNA, miRNAs, and long non-coding RNA to confirm any potential biomarkers that could find potential use in the detection and prognosis of AIS, as well as for the monitoring of AIS progression and prognosis along with endovascular treatment. Furthermore, in the NCT03577093 trial, the molecular mechanisms of microRNA-494 to mediate cell cycle regulation after the ischemic episode of cerebral tissue will be evaluated, where the peripheral blood DNA samples from the eligible study group will be analyzed, and will include participants aged between 18–80 years of life with a stroke onset within 6 h [179].

Comparing the cell-based therapy with the treatment based on exosomes, it was noticed that similar substantial protective effects are present while a reduction in the potential tumorigenic and immunogenic side effects were also observed [188].

### 7.3. Other Treatment Perspectives

A better understanding of, and improvements in, DNA sequencing, along with the development of new methods for gene mutation correction in humans, has placed genetic analysis as well as gene therapy in the focus of stroke investigations. Moreover, for stroke treatment, based on its seriousness, gene transfer techniques could be applicable since it could be useful in the protection of neurons, reduction of infarct size, and improvement of function, based on experimental studies [189].

CNS injuries are usually associated with neuronal loss and scar formation in glia. Furthermore, it was found that the direct conversion of glial cells that are reactive into functional neurons can be mediated by NeuroD1 in the brains of adult mice. Zhang et al. investigated the potential technology of direct glial scar reversion to neural tissue in the cortex within a mouse model. To ectopically express a single neural transcription factor NeuroD1 in reactive astrocytes at the injured site, they applied an adeno-associated virus (AAV)-based gene therapy approach. After the direct reversal of reactive astrocytes into neurons in the injured area in situ, the astrocyte-to-neuron (AtN) conversion technology appears to be a promising mode of glial scar to neural tissue reversion without the introduction of exogenous cells. After the AtN conversion and regeneration of new neurons, along with the reduction of reactive astrocytes, they detected numerous favorable effects, including a reduction of microglia and neuroinflammation, a rebalancing of the neuron to astrocyte ratio, an increase of neuronal dendrites and synaptic density, and blood–brain barrier restoration. Therefore, in vivo cell conversion technology could provide a potential alternative approach for neural repair by reversing glial scar tissue back to neural tissue [190]. After the rodent stroke study, a new stroke model study in adult, non-human primates (NHPs) was conducted, with the group of researchers demonstrating a consistent finding that the gene therapy that is based on NeuroD1 might have a favorable potential in the regeneration of a large number of new neurons in the adult mammalian brains [191,192]. Furthermore, in Rhesus Macaque monkeys, the expression of a single neural transcription factor NeuroD1 in reactive astrocytes that are caused by an ischemic type of injury can convert them into neurons at the injury site. Following the in situ AtN conversion, a significant increase in the neuronal density at the injured area that was treated by the NeuroD1 was noticed, along with an increase of dendritic and synaptic markers. It should be stated that in addition to the reduction of microglia and macrophages after the treatment by the NueroD1, it was noticed that parvalbumin interneurons were significantly protected from ischemic injury in NeuroD1-infected areas. These results suggest that in vivo cell conversion technology, in concert with the regeneration of new neurons at the site of an injury, also have a potential for the amelioration of the microenvironment in the sense of being more neuro-protective and neuro-permissive [192].

Currently, the development of a neuroprotective treatment for the penumbra region within the therapeutic window is considered as a main fundamental and clinical interest for stroke therapy. The development of preventive gene therapy of ischemic stroke in at-risk patients is of particular interest. Markosyan and colleagues, for the first time, provided evidence of the beneficial effects of preventive triple gene therapy by delivering the combination of genes *vegf165*, *gdnf*, and *ncam1.* The simultaneous intrathecal delivery of adenoviral vectors carrying genes encoding vascular endothelial growth factor (VEGF), glial cell-derived neurotrophic factor (GDNF), and neural cell adhesion molecule (NCAM) or gene-engineered umbilical cord blood mononuclear cells (UCB-MC) overexpressing recombinant VEGF, GDNF, and NCAM, showed positive effects on the preservation and recovery of the brain in rats after stroke modeling. The obtained data might add an additional knowledge for ischemic stroke treatment as a novel, and potentially successful, approach [193].

## 8. Conclusions

If we attempt to summarize the role of different genetic contributors in the pathogenesis of pediatric stroke, it appears that the discovery of a single gene variant may not be sufficient to completely explain the stroke causality. The majority of cases have combined inherited and acquired risk factors, sometimes united with a known monogenic disorder underlying stroke in neonates and children. Subsequently, the analysis of several genotype combinations would be a more promising approach in patients’ stratification. It will facilitate further individualization in stroke prevention, neuroprotection, repair-based therapies, and patient-tailored secondary prevention.

The focal point of this review was the importance of a wider perception in the conceptualization of pediatric stroke in order to not overlook any genetic aspect (monogenic, polygenic, risk factors, modifiers, etc.). As it is a multifactorial condition encompassed of several distinctive subtypes, every genetic variant, or perhaps, combination of several variants, may become a crucial clue in drug discovery that will provide a broad impact on stroke in infants and children. 

## Figures and Tables

**Table 1:** The most significant monogenic conditions associated with pediatric stroke, organized by mode of inheritance.

Disease Name	Gene Symbol	RefSeq ID	OMIM Gene	OMIM Clinical Phenotype
**Autosomal Dominant Inheritance**				
CARASAL Cathepsin A-related arteriopathy with strokes and leukoencephalopathy	*CTSA* (single variant R325C)	NM_000308.4	613111	MIM number not assigned yet
Autosomal dominant familial porencephaly	*COL4A1*	NM_001845.5	120130	175780
PADMAL Pontine autosomal dominant microangiopathy and leukoencephalopathy	*COL4A1* (3′-UTR)	NM_001845.5	120130	611773
CADASIL Cerebral autosomal dominant arteriopathy with subcortical infarcts and leukoencephalopathy	*NOTCH3*	NM_000435.2	600276	125310
ACTA2-related vasculopathy	*ACTA* (multiple variants at position R179)	NM_001141945.2	102620	611788 614042 613834
Marfan syndrome	*FBN1*	NM_000138.5	134797	154700
EDS-IV Ehlers-Danlos Syndrome type IV	COL3A1	NM_000090.4	120180	130050
HGPS Hutchinson-Gilford Progeria Syndrome	*LMNA*	NM_170707.4	150330	176670
Von Hippel–Lindau disease	*VHL*	NM_000551.4	608537	193300
NF-1 Neurofibromatosis Type 1	*NF1*	NM_001042492.2	613113	162200
NF-2 Neurofibromatosis type 2	*NF2*	NM_000268.4	607379	101000
AGS Acardi-Goutieres Syndrome 1, dominant	*TREX1*	NM_033629.6	606609	225750
**Autosomal Recessive Inheritance**				
SCD Sickle cell disease	*HBB* (β^S^ allele)	NM_000518.5	141900	603903
Grange syndrome	*YY1AP1*	NM_139119.3	607860	602531
TTP Thrombotic thrombocytopenic purpura	*ADAMTS13*	NM_139027.6	604134	274150
ADAMTS13 thrombotic microagiopathy	*ADAMTS13*	NM_139027.6	604134	MIM number not assigned yet
CARASIL Cerebral autosomal recessive arteriopathy with subcortical infarcts and leukoencephalopathy	*HTRA1*	NM_002775.4	602194	616779 600142
DADA-2 Deficiency of adenosine deaminase 2	*CECR1*	NM_001282225.2	607575	182410
PXE Pseudoxanthoma elasticum	*ABCC6*	NM_001171	603234	264800 177850
EDS-VIa Ehlers-Danlos syndrome type VIA	*PLOD1*	NM_000302.4	153454	225400
AGS Acardi-Goutieres Syndromes	*RNASEH2A* *RNASEH2B* *RNASEH2C* *SAMHD1* *TREX1*	NM_006397.3 NM_024570.4 NM_032193.4 NM_015474.4 NM_033629.6	606034 610326 610330 606754 606609	610333 610181 610329 612952 225750
**X-Linked Inheritace**				
Menkes disease	*ATP7A*	NM_000052.6	300011	309400
Fabry disease	*GLA*	NM_000169.2	300644	301500
**Mitochondrial Inheritance**				
MELAS Mitochondrial myopathy, encephalopathy, lactic acidosis, and stroke syndrome	*MTTL1*	NA	590050	540000
**Somatic Mutations**				
SWS Struge–Weber syndrome	*GNAQ* (single variant c.548G>A; R183Q)	NM_002072.5	600998	185300

**Table 2:** Genetic risk factors most commonly studied in pediatric stroke.

Polymorphi-sm Name	Gene Symbol	RefSeq ID	Variant Position	Amino Acid (Codon) Position	SNP Database Number	Proposed Association	References
A1298C	*MTHFR*	NM_005957.5	c.1298A>C	p.Glu429Ala	rs1801131	No association	[113,114,115,116,117,118,119,120,121,122,123,124,125]
C677T	*MTHFR*	NM_005957.5	c.677T>C	p.Ile226Thr	rs1217691063	Increasing risk	[115,116,119,121,122,126,127,128,129]
*FV* Leiden	*F5*	NM_000130.5	c.1601G>T	p.Arg534Leu	rs6025	Increasing risk	[124,130]
G20210A	*F2*	NM_000506.5	c.*97G>A	N/A	rs1799963	Increasing risk	[113,115,116,117,118,120,124,131,132,133,134,135,136,137,138,139,140]
V34L	*F13A1*	NM_000129.4	c.103G>T	p.Val35Leu	rs5985	No association	[115,128,133,141,142]
PAI-1 4G/5G	*SERPINE1 promotor*	NG_013213.1	g.4333A>G	N/A	rs1799889	No association	[115,118,128,134,142,143,144,145,146]
HPA-1	*ITGB3*	NM_000212.3	c.176T>C	p.Leu59Pro	rs5918	Increasing risk	[115,118,122,128,130]
HPA-2	*GP1BA*	NM_000173.7	c.482C>T	p.Thr161Met	rs6065	No association	[128,130]
HPA-3	*ITGA2B*	NM_000419.5	c.2621T>G	p.Ile874Ser	rs5911	Lowering risk for AIS, increasing risk for CSVT	[128,130]
HPA-5	*ITGA2*	ENST 00000296585.10	c.1600G>A	p.Glu534Lys	rs1801106	No association	[128,130]
ApoE e2–4	*APOE*	NM_001302691.2	c.388T>C c.526C>T	p.Cys130Arg p.Arg176Cys	rs429358 rs7412	No association	[116,128,147]

N/A—not applicable.
